# Secretory Expression and Characterization of Two Hemicellulases, Xylanase, and β-Xylosidase, Isolated from *Bacillus Subtilis* M015

**DOI:** 10.1007/s12010-014-1219-1

**Published:** 2014-09-16

**Authors:** Alison L. Banka, Saadet Albayrak Guralp, Erdogan Gulari

**Affiliations:** Department of Chemical Engineering, University of Michigan, 3074 H.H.Dow Buil., 2300 Hayward St, Ann Arbor, MI 48109 USA

**Keywords:** Hemicellulose, Xylan, Xylanase, β-xylosidase, Xylooligosaccharides, *Bacillus subtilis*, Secretory expression, *Escherichia coli* JE5505

## Abstract

Microbial hydrolysis of lignocellulosic biomass is becoming increasingly important for the production of renewable biofuels to address global energy concerns. Hemicellulose is the second most abundant lignocellulosic biopolymer consisting of mostly xylan and other polysaccharides. A variety of enzymes is involved in complete hydrolysis of xylan into its constituent sugars for subsequent biofuel fermentation. Two enzymes, endo-β-xylanase and β-xylosidase, are particularly important in hydrolyzing the xylan backbone into xylooligosaccharides and individual xylose units. In this study, we describe the cloning, expression, and characterization of xylanase and β-xylosidase isolated from *Bacillus subtilis* M015 in *Escherichia coli*. The genes were identified to encode a 213 amino acid protein for xylanase (glycoside hydrolase (GH) family 11) and a 533 amino acid protein for β-xylosidase (GH family 43). Recombinant enzymes were produced by periplasmic-leaky *E. coli* JE5505 and therefore secreted into the supernatant during growth. Temperature and pH optima were determined to be 50 °C and 5.5–6 for xylanase and 35 °C and 7.0–7.5 for β-xylosidase using beech wood xylan and p-nitrophenyl-β-D-xylopyranoside as the substrates, respectively. We have also investigated the synergy of two enzymes on xylan hydrolysis and observed 90 % increase in total sugar release (composed of xylose, xylobiose, xylotriose, and xylotetraose) for xylanase/β-xylosidase combination as opposed to xylanase alone.

## Introduction

Concerns about greenhouse gases, a finite fossil fuel supply, and an increase in energy needs around the world has led to an increased interest in alternative fuel sources such as the use of lignocellulosic biomass for biofuel production [1]. Several groups employed metabolic pathway engineering tools to modify microorganisms to convert processed lignocellulosic biomass to a renewable fuel source such as ethanol, biodiesel, and isobutanol [2–5]. Lignocellulosic biomass consists of three major components: cellulose (30–50 % dry weight), hemicellulose (20–40 %), lignin (15–25 %), and ash and other components (3–10 %) [6]. While both cellulose and hemicellulose can be hydrolyzed to individual sugar molecules, hemicellulose’s short, branched, non-crystalline nature makes it more ideal for hydrolysis than cellulose [7]. Hemicelluloses are comprised of several monosaccharide units, mainly xylan with lesser amounts of arabinan, galactan, and mannan, depending on the origin. The main hemicellulose of hardwoods and many agricultural crops is xylan, which is a linear polysaccharide consisting of D-xylose units linked by β-1,4-glycosidic bonds with a large variety of substitutions [8]. At least two enzymes, *endo*-1,4-β-xylanase (EC 3.2.1.8) and 1,4-β-xylosidase (EC 3.2.1.37), are required to hydrolyze the xylan backbone to individual xylose units, with additional enzymes required to cleave any side groups [9]; this hydrolysis allows an organism to use hemicellulose as a food source, providing xylose to be utilized as a sugar source [10].

Many xylanases have been previously isolated and characterized from different strains of bacteria and fungi [11–13]; β-xylosidases and the co-activity of two enzymes on xylan hydrolysis however have been described to a lesser degree [14–16]. In this study, we have cloned two genes coding for endo-xylanase and β-xylosidase from a recently-isolated strain *Bacillus subtilis* M015 and strategically expressed them in a periplasmic-leaky *Escherichia coli* strain which resulted in successful secretion of the recombinant proteins outside of the cells at high levels. The two enzymes were characterized separately, and both their individual and combined effect on hydrolysis of beech wood xylan was examined. Secretory expression of endo-xylanase and β-xylosidase allows them to be readily available in culture supernatant and continuously hydrolyze xylan into xylose and other xylooligosaccharides which can be consequently utilized by an engineered fuel-producer strain to generate biofuels.

## Materials and Methods

### Microbial Strains and Plasmids

*B. subtilis* M015 was previously isolated from Thai higher termites, *Microcerotermes* sp., [17] and provided by Professor Sumaeth Chavadej’s Lab at the Petroleum and Petrochemical College at Chulalongkorn University in Bangkok, Thailand. *E. coli* JE5505 (carrying an *lpp*-deletion) was obtained from The Coli Genetic Stock Center (CGSC) at Yale University and the plasmid pFLAG-CTS was purchased from Sigma-Aldrich.

### Chemicals, Substrates, and Media

*B. subtilis* M015 was grown in tryptic soy broth (TSB), and *E. coli* JE5505 was maintained in lysogeny broth (LB). *p*-nitrophenol (*p*NP) and *p*-nitrophenyl-β-D-xylopyranoside (pNPX) were purchased from Acros Organics and Calbiochem, respectively. Xylooligosaccharides 1,4-β-D-xylobiose, 1,4-β-D-xylotriose, and 1,4-β-D-xylotetraose (Megazyme) were provided by Professor Xiaoxia Lin’s Lab (University of Michigan) to be used as standards (X1 to X4) for high-performance liquid chromatography (HPLC).

### Cloning and Expression of Recombinant Endo-Xylanase and β-Xylosidase

The following two primers were used for the amplification of the xylanase gene by polymerase chain reaction (PCR) using *B. subtilis* M015 genomic DNA as the template: forward primer (*HindIII*) 5′-CAGGATCC**AAGCTT**CTATGTTTAAGTTTAAAAAGAATTTCT-3′, reverse primer (*EcoRI*) 5′-GCTCA**GAATTC**TT ACCACACTGTTACGTTA-3′. The xylanase gene was amplified using Phusion High-Fidelity DNA Polymerase (New England Biolabs) with the following PCR program 7 min at 98 °C, 30 s at 98 °C, 30 s at 53 °C, and 30 s at 72 °C (33 cycles), and 5 min at 72 °C. Amplification of the β-xylosidase gene was performed similarly using the following primers: forward primer (*XhoI*) 5′-GTA**CTCGAG**AAATGAAGATTACCAATCCC-3′ and reverse primer (*EcoRI*) 5′-GCC**GAATTC**TTATTTTTCTTTATAACGAAAATATC-3′. Both primer sets were designed based on the sequence of xylanase (*xynA*) and β-xylosidase (*xynB*) genes of *B. subtilis* 168 (GenBank accession number; AL009126.3). Standard molecular biology techniques were performed for the purification, digestion, and ligation of the PCR products and the linearized plasmid DNA. Ligation products (pCTS-XynA and pCTS-XynB) were transformed into *E. coli* JE5505 by electroporation, and the cloning of the genes was confirmed by DNA sequencing.

For recombinant enzyme production, *E. coli* cells carrying either pCTS-XynA or pCTS-XynB were grown overnight in 2 mL LB-Ampicillin (100 μg/mL), and diluted 1:400 in 10 mL M9 minimal media containing 100 μg/mL ampicillin. Once the optical density of the culture reached OD_600_ = 0.5, gene expression was induced with 1 mM of isopropyl-β-D-thiogalactopyranoside (IPTG, Invitrogen Life Technologies) for 16 h at 37 °C. Next day, the cells were pelleted at 4 °C and clarified supernatant was sterilized using a 0.22 μm filter. This supernatant was used as crude enzyme (visualized by sodium dodecyl sulfate-polyacrylamide gel electrophoresis) in the following activity assays.

### Enzyme Assays

#### Assay for Xylanase Activity

Endo-1,4-β-xylanase activity was determined by the dinitrosalicylic acid (DNS) method [18]. Briefly, 1 % beech wood xylan was dissolved in 50 mM sodium citrate buffer at pH 6.0 and incubated with diluted crude enzyme for 10 min at 50 °C. An equal volume of DNS solution (1.4 % 3,5-dinitrosalicyclic acid, 0.28 % phenol, 0.07 % sodium sulfite, 28 % sodium potassium tartrate, 1.4 % sodium hydroxide) was added to each sample and incubated at 95 °C for 5 min. The absorbance of the mixture was measured at 540 nm, and amount of sugars released from xylan were calculated using D-xylose as a standard. The specific activity (IU/mL) of XynA is defined as the number of micromoles of xylose released per minute per unit volume of XynA crude enzyme. Relative activity of XynA for the temperature profile was determined by dividing the specific activity of XynA at a given temperature by that at 50 °C. The relative activity of XynA for the pH profile was determined by dividing the specific activity at a given pH by that at a pH 5.5 in sodium citrate buffer.

#### Assay for β-Xylosidase Activity and End-Product Inhibition by Xylose

1,4-β-xylosidase activity was assessed by “pNPX assay” through incubation of the crude enzyme in 50 mM sodium phosphate buffer at pH 7.0 with 5 mM *p*-nitrophenyl-β-D-xylopyranoside (pNPX) in buffer. The incubation took place at 40 °C for 10 min in a VersaMax microplate reader (Molecular Devices) measuring absorbance at 410 nm every 15 s. The specific amount of *p*-nitrophenol (pNP) released was calculated using pNP standards at varying concentrations. The specific activity (IU/mL) of XynB is defined as the micromoles of pNP released per minute per unit volume of XynB crude enzyme. Relative activity of XynB for the temperature profile was determined by dividing the specific activity of XynB at a given temperature by that at 35 °C and the relative activity of XynB for the pH profile was determined by dividing the specific activity at a given pH by that at a pH 7.5 in sodium phosphate buffer.

To investigate the inhibitory effect of released xylose on XynB activity, increasing concentrations of xylose (up to 150 mM) in 50 mM sodium phosphate buffer at pH 7.0 was added to each well in the pNPX assay. The relative activity of XynB at different concentrations of xylose was calculated by dividing the specific activity at a given concentration of xylose by that of control reaction without xylose.

#### Effect of pH and Temperature on Activity

The optimal pH for XynA was determined by performing the DNS assay in 50 mM sodium citrate buffer for pH 4.5–6.0 and 50 mM sodium phosphate buffer for pH 6.0–8.0. The optimal temperature for XynA was determined in 50 mM sodium citrate buffer (pH 6.0) at temperatures ranging from 30 to 70 °C. The optimal pH for XynB was determined by pNPX assay using the same buffer and pH range that was used for XynA. The optimal temperature for XynB was determined in 50 mM sodium phosphate buffer (pH 7.0) in temperatures ranging from 22 to 70 °C.

#### Synergy of Xylanase and β-Xylosidase on Xylan Hydrolysis

The synergism between XynA and XynB was investigated by combining crude enzymes in 1:1, 1:2, and 1:3 ratios with an equal volume of 1 % beech wood xylan prepared in 50 mM sodium phosphate buffer (pH 7.0). A reference reaction was also set up in a similar way using XynA alone. The mixture was incubated at 40 °C for 16 h and the collected samples were examined by HPLC (1100 series, Agilent Technologies) with a RAO organic acid column. 0.0025 M H_2_SO_4_ was used as the mobile phase, 5 μL of each sample was injected into the system with a flow rate of 0.3 mL/min, and the column was run at 60 °C.

## Results and Discussion

### Cloning and Expression of Recombinant Xylanase and β-Xylosidase

Amplification of xylanase and β-xylosidase genes from *B. subtilis* M015 using specific primer pairs yielded 642 and 1602 bp DNA fragments, respectively. Complete sequencing of both strands of each fragment revealed that these genes are encoding for a protein of 213 amino acid residues for XynA (calculated MW of 23,358 Da) and a protein of 533 amino acid residues for XynB (calculated MW of 61,365 Da). The amino acid sequence of XynA was 100 % identical to that of *B. subtilis* R5 (GenBank; AB457186) and 99 % identical to that of *B. subtilis* subsp. *subtilis* 168 (AL009126.3, GeneID; 939861). The amino acid sequence of XynB was 100 % identical to that of *B. subtilis* subsp. *subtilis* 168 (AL009126.3, GeneID; 939472) and 99 % identical to that of *B. subtilis* BSP1 (GenBank, CP003695). These findings confirm that XynA belongs to the glycoside hydrolase (GH) family 11 whereas XynB belongs to GH family 43.

**Fig. 1:**
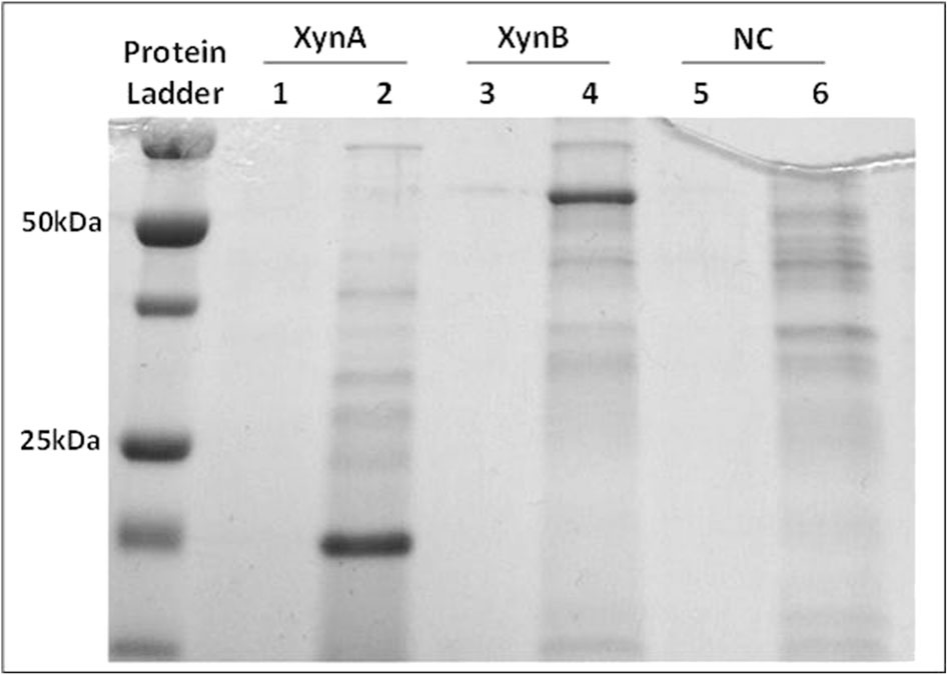
SDS-PAGE of recombinant enzymes. *Lanes 2 and 4* show dominant blue protein bands present in culture supernatants corresponding to recombinant XynA and XynB, respectively. ‘*NC*’ corresponds to *E. coli* JE5505 cells containing only pFLAG-CTS. Supernatant samples loaded to *lanes 1, 3, and 5* were taken before inducing protein expression

The maximum activities of the crude enzymes were determined as 2.75 ± 0.30 IU/mL and 0.41 +/−0.02 IU/mL for XynA and XynB, respectively. Both XynA, which was purified and concentrated using centrifugal filters (Millipore), and XynB were visualized on a 12 % sodium dodecyl sulfate polyacrylamide gel electrophoresis (SDS-PAGE) gel stained with Coomassie Blue (Biorad) and had molecular weights of approximately 20 and 60 kDa, respectively (Fig. 1), consistent with their theoretical molecular weights.

### Xylanase Enzymatic Characteristics

**Fig. 2:**
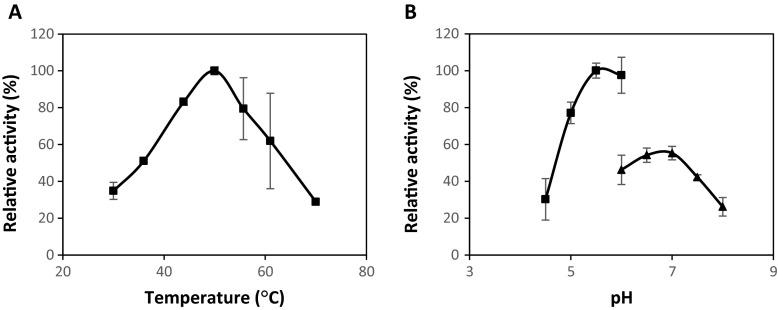
Temperature and pH profiles of recombinant XynA. **a**. Effect of temperature on enzyme activity measured at pH 6.0 in sodium citrate buffer (*filled squares*). **b**. Effect of pH on enzyme activity measured at 50 °C using sodium citrate buffer and sodium phosphate buffer (*filled triangles*). *Error bars* represent standard deviation of triplicate assays

The characteristics of the recombinant xylanase were determined using beech wood xylan as the substrate. The temperature and pH profile of XynA can be found in Fig. 2a, b. As with many xylanases previously described, XynA prefers mesophilic and slightly acidic conditions. Under the assay conditions used in our study, XynA displayed optimum activity at 50 °C while retaining over 50 % relative activity between 37 and 60 °C. Unlike other xylanases of *B. subtilis* CXJZ [19] and *B. subtilis* cho40 [13], XynA has a lower optimum temperature, making it very similar to xylanases of *B. subtilis* B-10 [20] and *B. subtilis* R5 [21] whose sequence is identical to XynA (optimum temperature reported as 40 to 50 °C). XynA performed optimally in sodium citrate buffer at pH 5.5–6.0; however, its activity quickly dropped below pH 5.0 and when in sodium phosphate buffer of any pH. The activity profile of XynA in sodium citrate buffer is consistent with xylanases from strains mentioned above including *B. subtilis* R5; however, unlike others, it displays much lower activity in phosphate buffer.

### β-Xylosidase Enzymatic Characteristics

**Fig. 3:**
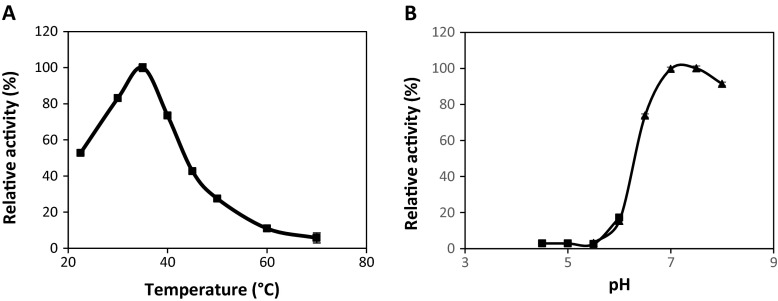
Temperature and pH profiles of recombinant XynB. **a**. Effect of temperature on enzyme activity measured at pH 7.5 in sodium citrate buffer (*filled squares*). **b**. Effect of pH on enzyme activity measured at 35 °C using sodium citrate buffer and sodium phosphate buffer (*filled triangles*). *Error bars* represent standard deviation of triplicate assays

We have characterized the xylosidase activity of recombinant XynB using pNPX as the substrate and its temperature and pH profiles are shown in Fig. 3a, b. The optimum temperature for XynB was determined as 35 °C. Its activity at 40 °C, often the optimum temperature for GH43 β-xylosidases isolated from bacteria, drops to around 75 % relative to its activity at 35 °C. The temperature used to determine xylosidase activity varies greatly in literature for β-xylosidases of *Bacillus spp*.; 25 °C for *B. subtilis* subsp. *subtilis* 168 [22], 30 °C for *B. subtilis* PAP115 [23], and 50 °C for *B. subtilis* (subspecies not reported) [24]. Therefore, XynB’s optimum temperature falls within the range of closely-related β-xylosidases. Similar to its temperature profile, XynB’s optimum pH of 7.0 agrees with the pH profile of most GH-43 β-xylosidases [22–24]. XynB showed highest activity in sodium phosphate buffer at pH 7.0–7.5 but dropped to a relative activity of around 20 % at pH 6.0. Interestingly, we have observed that, besides its xylosidase activity, XynB also displays lower galactosidase activity when tested with 2-nitrophenyl-β-galactopyranoside (oNPGal) but no glucosidase activity was detected when tested with 4-nitrophenyl- β-glucopyranoside (pNPG) (unpublished results).

**Fig. 4:**
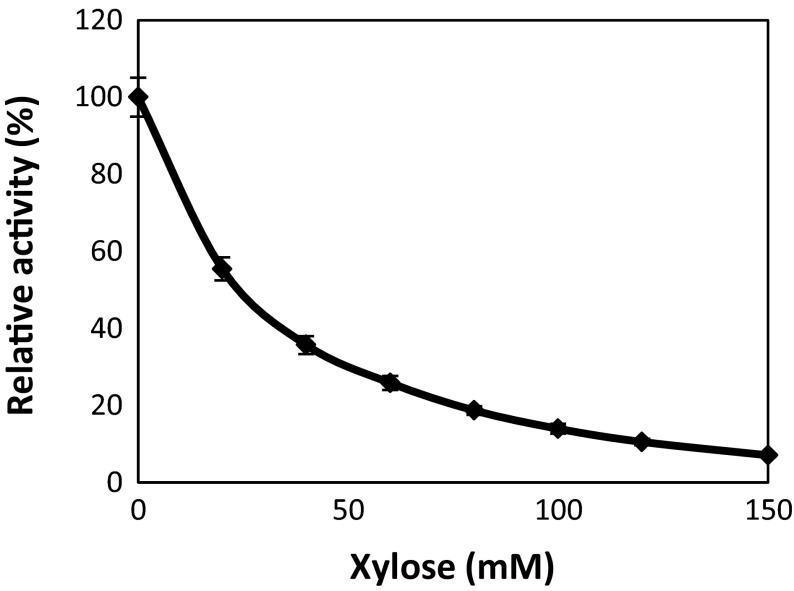
Inhibition of XynB by xylose. *Error bars* represent standard deviation of triplicate assays

Previous studies have shown that some β-xylosidases experience end-product inhibition by xylose present in the reaction mix [15, 25]. We have observed a similar effect on XynB where its relative activity quickly dropped to 55 % in the presence of 20 mM of xylose and continued decreasing with increasing xylose concentration in the reaction mix (Fig. 4). However, this should not present an issue for consolidated bioprocesses with fuel-producer strains wherein xylose is consumed before it accumulates to inhibitory concentrations.

### Synergy of Xylanase and β-Xylosidase on Xylan Hydrolysis

**Fig. 5:**
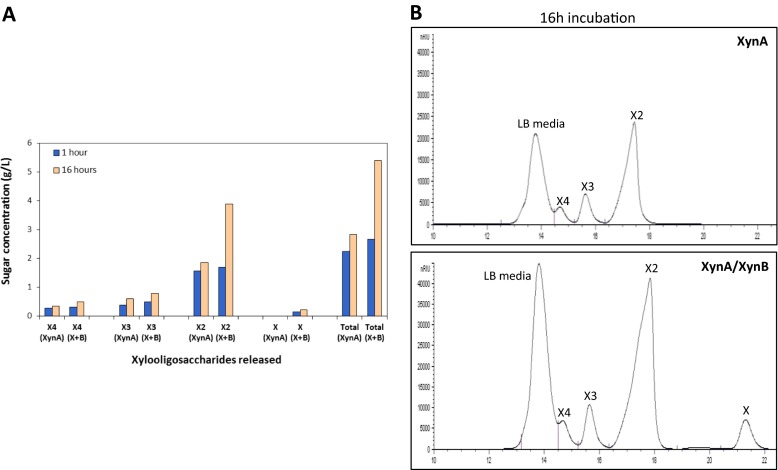
Xylan hydrolysis by XynA and XynA/XynB (at 1:3 ratio) **a**. Amount of different sugars released at 1 and 16 h incubation (determined by DNS assay) **b**. HPLC diagram of xylan hydrolysis by XynA (*top*) and XynA/XynB (*bottom*) after 16 h incubation. *X + B* XynA + XynB, *X4* xylotetrose, *X3* xylotriose, *X2* xylobiose, and *X* xylose

To investigate the co-activity of XynA and XynB, varying ratios of two enzymes were incubated with beech wood xylan and produced oligosaccharide profiles were determined by HPLC. When tested alone, XynA released mostly xylobiose and to a lesser amount, xylotriose and xylotetraose; but no xylose was detected in the samples. The combination of XynA and XynB, however, allowed for the production of xylose as well as increased amounts of all xylooligosaccharides examined (xylose, xylobiose, xylotriose, and xylotetraose) as seen in Fig. 5.

In particular, combining the two enzymes in a 1:3 volume ratio released the most sugar from xylan over 16 h incubation. After an incubation of only 1 h, the XynA/XynB combination increased the total amount of sugars released by about 20 % with xylobiose being the primary sugar released. By 16 h, the total amount of sugars released was about 90 % higher than that of XynA alone again with the primary sugar as xylobiose. A similar synergistic effect was noticed by Carapito et al. (2009), Bao et al. (2012), and Zhou et al. (2012) who also reported that the combination of the two enzymes led to xylose formation with an excess of xylobiose [16, 26, 27]. It should be noted here that, although a standard for xylopentaose was not included in this study, the increase in the peak for LB media indicates that the hydrolysis of xylan with XynA/XynB complex leads to formation of another product, possibly xylopentaose, whose peak being overlapped by that of growth media.

In conclusion, we have successfully isolated and expressed the genes encoding for endo-xylanase and β-xylosidase of *B. subtilis* M015 in *E. coli* JE5505 and characterized their activity in xylan hydrolysis. The compatibility of optimum reaction conditions and their consequently high synergistic activity makes them compatible with bacterial cultivation and will allow these clones to be used in a consolidated bioprocess with a fuel-producer strain to convert xylooligosaccharides into biofuels (i.e., ethanol, isobutanol, etc.) by using xylan as the feedstock.
